# Using policy codesign to achieve multi-sector alignment in adolescent behavioral health: a study protocol

**DOI:** 10.1186/s43058-024-00583-y

**Published:** 2024-05-08

**Authors:** Sarah Cusworth Walker, Kym R. Ahrens, Mandy D. Owens, McKenna Parnes, Joe Langley, Christine Ackerley, Jonathan Purtle, Lisa Saldana, Gregory A. Aarons, Aaron Hogue, Lawrence A. Palinkas

**Affiliations:** 1 Department of Psychiatry and Behavioral Sciences, University of Washington, Seattle, USA; 2 Seattle Children’s Hospital and Research Institute, Seattle, WA USA; 3 Addictions, Drug & Alcohol Institute, University of Washington, Seattle, WA USA; 4 Lab For Living, Sheffield Hallam University, Sheffield, UK; 5 Department of Public Health Policy and Management, Global Center for Implementation Science, New York University, New York City, USA; 6 Chestnut Health Systems, Lighthouse Institute, Eugene, OR USA; 7 Herbert Wertheim School of Public Health and Human Longevity Science, University of California San Diego, La Jolla, CA USA; 8 Department of Psychiatry, UC San Diego ACTRI Dissemination and Implementation Science Center, University of California San Diego, La Jolla, CA USA; 9 Partnership to End Addiction, New York, NY USA

**Keywords:** Policy design, Formation, Evidence use, Adolescent, Substance use, Behavioral health

## Abstract

**Background:**

Policymaking is quickly gaining focus in the field of implementation science as a potential opportunity for aligning cross-sector systems and introducing incentives to promote population health, including substance use disorders (SUD) and their prevention in adolescents. Policymakers are seen as holding the necessary levers for realigning service infrastructure to more rapidly and effectively address adolescent behavioral health across the continuum of need (prevention through crisis care, mental health, and SUD) and in multiple locations (schools, primary care, community settings). The difficulty of aligning policy intent, policy design, and successful policy implementation is a well-known challenge in the broader public policy and public administration literature that also affects local behavioral health policymaking. This study will examine a blended approach of coproduction and codesign (i.e., Policy Codesign), iteratively developed over multiple years to address problems in policy formation that often lead to poor implementation outcomes. The current study evaluates this scalable approach using reproducible measures to grow the knowledge base in this field of study.

**Methods:**

This is a single-arm, longitudinal, staggered implementation study to examine the acceptability and short-term impacts of Policy Codesign in resolving critical challenges in behavioral health policy formation. The aims are to (1) examine the acceptability, feasibility, and reach of Policy Codesign within two geographically distinct counties in Washington state, USA; (2) examine the impact of Policy Codesign on multisector policy development within these counties using social network analysis; and (3) assess the perceived replicability of Policy Codesign among leaders and other staff of policy-oriented state behavioral health intermediary organizations across the USA.

**Discussion:**

This study will assess the feasibility of a specific approach to collaborative policy development, Policy Codesign, in two diverse regions. Results will inform a subsequent multi-state study measuring the impact and effectiveness of this approach for achieving multi-sector and evidence informed policy development in adolescent SUD prevention and treatment.

Contributions to the literature

- This study will illuminate the feasibility of blending community-engaged and evidence-informed approaches to behavioral health policymaking.
- This study will assess the impact of adapting policymaking processes to align with the cultural and economic diversity of different regions.
- Findings from this study will advance efforts to define the appropriate measures for assessing successful evidence-informed policymaking.

## Introduction

Serious adolescent substance use disorder (SUD) continues to affect over one million adolescents and young adults a year in the USA and disproportionately impacts individuals living with co-occurring serious mental health needs and poverty [1]. In the era of fentanyl and other synthetic opioids, consequences of use are growing more dire and include increased emergency room visits, hospitalizations, and fatal or near fatal overdoses [2–6]. These consequences disproportionately affect youth of color, particularly American Indian/Alaskan Native and Latinx Adolescents [2].

Policymaking is quickly gaining focus in the field of implementation science as a potential opportunity for aligning cross-sector systems and introducing incentives to promote population health, including SUD and its prevention in adolescents [7, 8]. Policymakers are seen as holding the necessary levers for realigning service infrastructure to address adolescent behavioral health more rapidly and effectively across the continuum of need (prevention through crisis care) and in multiple locations (schools, primary care, community settings) [9].

Local public policy tools include multiple options for addressing adolescent substance misuse through outer system context “big P” policies that leverage tax, regulation and other legal avenues, and inner organizational context “small p” policies that focus on organizational, coalition, and community-based initiatives [10–13]. Although there is great potential for policy to be leveraged to improve population health, studies of behavioral health policy implementation suggest that policy design will have to anticipate and overcome barriers such as low political motivation [14], limited human and financial resources [15–17], limited intersectoral collaboration [7], and suboptimal use of research evidence [14, 18–20].

### Policy and implementation science

The difficulty of aligning policy intent, policy design, and successful policy implementation is a well-known challenge in the broader public policy and public administration literatures that also affects local behavioral health policymaking [17, 21]. While policy studies are beginning to identify promising strategies for overcoming barriers to policymakers’ engagement with research evidence [13, 22–24], the field lacks an empirically supported approach to behavioral health policy design that anticipates barriers to policy implementation. An effective approach to local behavioral health policy design for adolescent SUD would need to demonstrate improvements across domains such as (a) multisector collaboration; (b) use of research evidence; (c) political will to address adolescent SUD; and (d) identification and commitment to use existing resources (e.g., financial and human) to support policy implementation.

Multiple scholarly areas (e.g., policy science, public administration research, health policy research) are exploring how to improve policy design to support successful downstream implementation [25, 26]. Two scholarly areas are particularly influential in the methods informing this study: Coproduction and codesign. Coproduction is a public policy concept used to describe processes aimed at improving alignment between citizen and government service delivery sectors through participatory processes [27–29]. As a conceptual model, coproduction views policy formation as a critical opportunity/window to proactively address potential policy implementation problems. Coproduction uses strategies to increase shared ownership of a policy’s design among policymakers and multisector coalitions of service delivery providers, non-government organizations, and service users/residents.

**Fig. 1:**
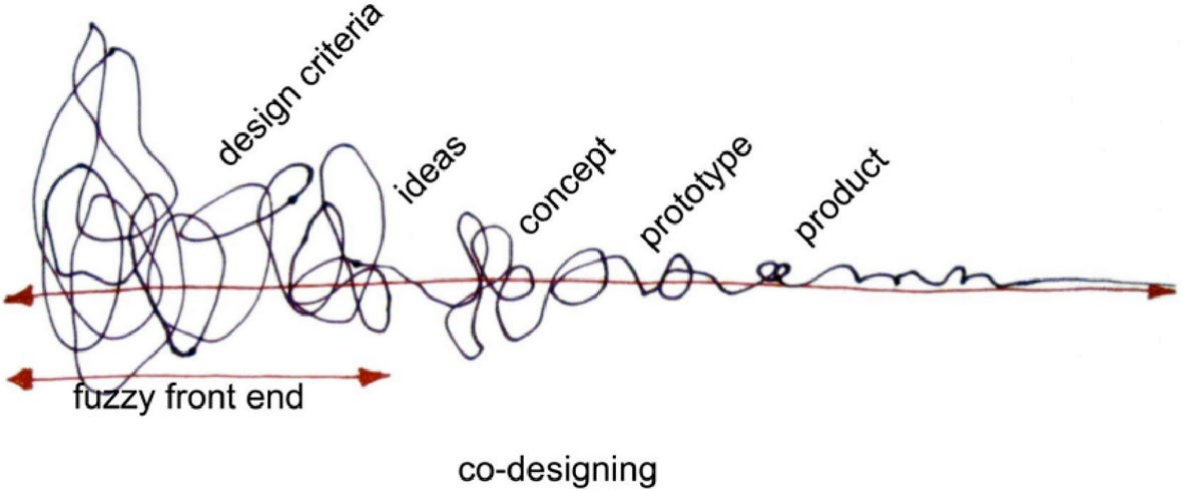
Taken from Sanders EB-N, Stappers PJ. Co-creation and the new landscapes of design. Codesign [Internet] 2008; 4(1):5–18. Available from: http://dx.doi.org/10.1080/15710880701875068

Codesign, as articulated in the field of design and design-thinking [30], as defined by Sanders and Stappers, is “the creativity of designers and people not trained in design working together in the design development process” (pg. 2). The field of codesign provides methods of information sharing and sense-making not as clearly structured or articulated in coproduction and, as a result, lends itself more readily to replication and testing. Codesign methods also typically follow a time-bound, linear process with nonlinear activities within phases that move codesigners from high uncertainty about how to solve a problem to more agreement among participants over time. This linear-nonlinear, time-bound approach is well-suited for policy formation which often occurs in policymaking windows (e.g., in anticipation of a legislative session) while allowing for new perspectives and new information to shift the direction of inquiry and planning as part of the process (Fig. 1).

The integration of codesign within coproduction is an emerging method in implementation science [31], with qualitative and case study research suggesting that it improves policy implementation across varying areas of health [32–34]. However, the field lacks quantitative analysis of the impacts of codesign and coproduction efforts on behavioral health policy design. A mixed methods evaluation can advance the field’s understanding of this integrated approach to improve behavioral health policymaking.

### The EPIS framework

The Exploration, Preparation, Implementation, and Sustainment (EPIS) framework is increasingly used to understand policy implementation failures in behavioral health [35, 36]. A recent study [35] of four states’ experiences with implementing a complex evidence-based practice found that policy adoption (e.g., policymaker-mandated use) was insufficient for ensuring fidelity to the intervention despite sufficient funding for training and program reimbursement and the use of 29 different implementation strategies. A critical implementation barrier for these states was the mismatch in “mindset” between service providers and the required evidence-based practice - that is, providers did not have the requisite attitudes and preparation needed for uptake of the new practices. This led to poor fit between the requirements of the policy and local context including the perceived fit of the intervention by providers. To overcome this challenge, the study authors recommended paying more attention to “pre-implementation” strategies, of which policy codesign is one example. EPIS is useful to understand relationships between legislative policies (outer context “big P” policies), downstream health insurer benefit design (outer context “little p” policies) and their effects on patient access to care in the inner context of healthcare organizations (inner context “little p” policies [37]. EPIS also identifies the importance of “bridging factors” that represent bi-directional influences of inner context people and organizations (e.g., advocacy) with outer context people and entities engaged in the development and enactment of policies [38, 39].

Stronger pre-implementation planning is expected to avoid these implementation failures and facilitate implementation success by (1) aligning outer context policy with service sector preparation and (2) aligning capacity and inner context services to meet community needs, and (3) being driven by research evidence [40]. Integrating coproduction with the EPIS implementation framework as a guiding theory provides an evaluative framework for determining whether policy solutions are likely to achieve successful, downstream implementation and long-term public health improvement.

### Current study

This study examines Policy Codesign, a blended approach of coproduction and codesign that was iteratively developed over multiple years to address problems in policy formation related to poor implementation outcomes [41, 42]. Two published studies found that early versions of this approach were successful in achieving cross-sector policy changes in little and big “P” policies, respectively [41, 42]. In the first study, a policy codesign process resulted in a cross-sector memorandum of understanding and implemented a program for adult jail-based medications for opioid use disorder and reentry support services within eight months of beginning the design intervention [42]. A second study demonstrated the acceptability and feasibility of community policy ranking sessions as a strategy for engaging democratic participation in selecting evidence-informed policy strategies [41]. A recent scoping review of policy codesign, published in *Implementation Science*, identifies this as a growing area of interest in health services research, political science, and design research fields [31]. However, research in this area is dominated by case studies, making the assessment of different approaches difficult.

The current study evaluates a scalable approach using reproducible measures in an effort to grow the knowledge base in this field of study. Given the accelerating pace of severe adolescent SUD consequences (e.g., overdose rates), the potential impact of successful multisector collaboration on evidence-informed behavioral health policymaking [7], and increasing interest in policy-focused implementation science [38, 43–47], the current study will make a timely contribution to advance policy-focused implementation science as well as local government approaches to preventing adolescent substance misuse.

## Methods

Using a single-arm, longitudinal with staggered implementation design, the aims of the current study are to (1) examine the acceptability and feasibility of Policy Codesign in resolving critical challenges in behavioral health policy formation within two geographically distinct counties in Washington state, USA; (2) examine the impact of Policy Codesign on multisector policy development within these counties; and (3) assess the perceived replicability of Policy Codesign among leaders and other staff of policy-oriented state behavioral health intermediary organizations.

### Study sites

The two study counties are different in geography, economics, and population density, and were selected to demonstrate proof of concept for Policy Codesign as an implementation strategy in diverse settings. King County is primarily urban, with a densely populated core (2.3 million residents in 2020). Okanogan County is rural and has a low population density (42,000 residents in 2020). King County is more racially and ethnically diverse than Okanogan County. Both counties have approved a 0.1% sales tax increase to raise funds for behavioral health services.

### Participants

Eligible study participants will be drawn from three levels of involvement in the Policy Codesign process: (a) core codesign team members, (b) advisory team groups, and (c) community sounding board. Policy Codesign *core design members* include individuals who participate in Policy Codesign sessions and directly contribute to the development of adolescent treatment planning. Core codesign team members will represent three vertical sectors: citizen/consumer, service delivery, and policy, with horizontal layers added to ensure the inclusion of all relevant actors in adolescent SUD services for each county. For example, a horizontal layer at the service delivery could be expanded to include schools, primary care, and housing. *Advisory team groups* will typically be 15 or more members of multi-sector partners that will meet regularly to assess acceptability and feasibility of emerging ideas and policy recommendations. *Community sounding board members* will be the largest sample of participants, with a target representation of 2% of the community affected by policy recommendations (i.e., households with children under 18 years) and >5% of adolescent SUD treatment and prevention service providers in each site. The community sounding board will be recruited through paid and targeted social media campaigns, project amplifying organizations, and focused requests through service provider listservs. The denominators for community and provider population will come from census data and the Washington state Department of Health, respectively [48].

### Recruitment

Participants for the codesign and advisory team groups will be identified from already committed county partners in each site. Participation in Policy Codesign activities will not be contingent on participating in research activities.

#### Purposeful selection

**Fig. 2:**
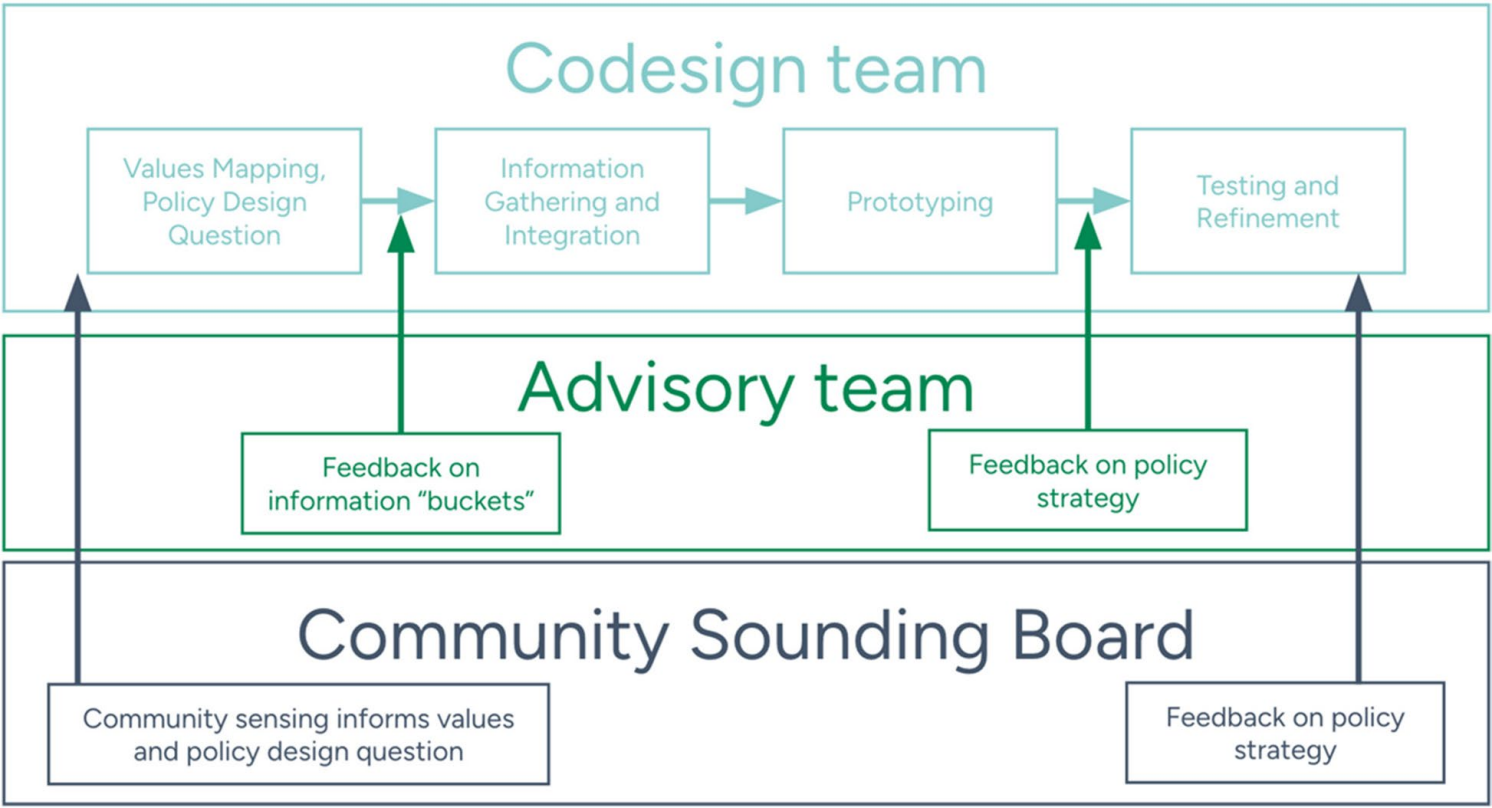
Purposeful selection

Using an approach informed by purposeful sampling [49], the county project team (i.e., research team and committed partners) will individually develop an initial list of potential partners within sector that represent three vertical levels (local government and policymakers, service sectors, community) and horizontal representation within levels across sectors relevant to adolescent SUD (public health, human services, education, advocacy, community coalitions); see Fig. 2.

#### Informational meeting

All members from the core and advisory Policy Codesign teams will be invited to an informational meeting about study research procedures prior to the start of the Policy Codesign intervention in their county. Individuals who do not attend will be emailed a link to a recording of the meeting and will have access to written resources describing research procedures.

#### Community sounding board

Community sounding board members will be recruited through paid social media, listservs, and membership lists managed by the codesign and advisory team organizational partners (project amplifiers) and are expected to include, for example, consumer advocacy groups, neighborhood associations, faith-based organizations, school to family communications, and adolescent SUD professional associations and individual invitations. All residents of the county sites will be eligible to join the sounding board. Individuals will be asked to share their zip code and neighborhood name before they are added to the sounding board. A standard message will be sent through these communications forums with a description of the initiative and an invitation to respond to a contact form if interested in being part of the community sounding board. Interested individuals will be contacted by the project team through an online community forum platform where they will be able to access project resources as well as receive push notifications through email. Participants will be entered into a lottery as an incentive for participation.

### Data collection

#### Aim 1: examine the acceptability and feasibility of Policy Codesign to develop adolescent SUD services in two diverse counties

The acceptability and feasibility of the Policy Codesign process will be assessed via attendance, surveys, focus groups, and individual interviews (see Table 1).

**Table 1:** Data collection plan

	Measure	Participant sample	Start	6 m	12 m
**Acceptability**					
Attendance	% attendance % completing out-of-session activities	Codesign and advisory members	Ongoing	Ongoing	
Satisfaction	Partnership synergy [31]	Codesign and advisory members		X	X
**Feasibility**					
Reach	% engagement/per capita			ongoing	
Perceived policy implementability	Policy concern [32]	Codesign and advisory members		X	X
**Multisector collaboration**		Community sounding board			
Sector cohesion, growth, and bridging	Social network analysis	Codesign and advisory members		Monthly	X
**Perceived replicability**	Focus group protocol	Policy intermediaries			X

One of the two acceptability outcomes will be attendance**,** defined by three metrics: (a) > 50% agreement to participate in Policy Codesign core and advisory roles following an invitation; (b) representation on the codesign and advisory teams reflecting policy, service and community sectors, at least 1–2 per sector on the core design team and 3–5 per sector on the advisory teams; and (c) active participation defined as participants attending > 70% of Policy Codesign scheduled meetings and 70% of participants engaging in self-guided, outside-of-meeting activities. The second acceptability outcome will be satisfaction assessed via surveys with core codesign and advisory teams, which will include the 7-item Partnership Synergy scale [50], at 6 and 12 months post-implementation.

The first feasibility outcome will be reached, defined as the proportion of county residents who provide input during the Policy Codesign process within household types stratified by race/ethnicity, household income, and professional sector. The second feasibility outcome will be perceived implementability as measured by the Policy Concern subscale [51], which will be included in 6- and 12-month surveys.

At 12 months following the start of Policy Codesign in each county, focus groups will be conducted with codesign core team members and advisory group members by study staff not participating in the Policy Codesign to encourage the sense of safety among members to speak freely about the process. The focus group protocols will draw from the measures used to assess satisfaction, partnership, collaboration, the resulting policy strategy, and likelihood of policy implementation. After focus groups, a subset of participants also will be invited to complete individual interviews. Individual interviews will aim to increase the richness of qualitative data regarding dissonance (divergent perspectives) or to gain a greater depth of understanding of common views expressed by focus group members that suggest Policy Codesign has problems with acceptability or policy feasibility. Accordingly, invitations for individual interviews will prioritize those who did not attend a focus groups, who had poor engagement during Policy Codesign (or refused an invitation to participate), and those who, in focus groups, offered useful insights into how Policy Codesign could be improved (or who found it unacceptable). Approximately 10–20 individual interviews will be completed across both sites until thematic saturation is reached.

#### Aim 2: examine the impact of Policy Codesign on multisector collaboration

Social network analysis (SNA) will be used to observe changes in multisector collaboration during and following Policy Codesign. SNA analysis will be informed by Valente et al.’s [52] recommendations for using SNA in implementation science research and will focus on changes in network size, cohesion, and bridging/brokering activities related to the developed adolescent SUD policy [52].

For the current study, SNA will examine the collaboration among multisector partners engaged in the Policy Codesign process who are part of either the codesign or advisory team. An online survey will be distributed via Qualtrics to Policy Codesign partners and stakeholders. Key quantitative metrics will be the number of *nodes* (Policy Codesign participants as well as individuals/organizations identified as key stakeholders for adolescent SUD policy); measures of *centrality* (the number of inter-connections within the network), including degree centrality (individuals/organizations with the greatest influence in the network) and betweenness (individuals/organizations who are bridges between nodes and control flow of information); and measures of *cohesion*, including reciprocity (formation of mutual relationships) and transitivity (formation of closely clustered relationships).

#### Aim 3: assess the perceived replicability of Policy Codesign with national adolescent SUD intermediary organizations

A qualitative analysis will be conducted on the perceived replicability of Policy Codesign among behavioral health policy intermediaries working with state and local behavioral health systems across the USA. In this aim, we will measure the perceived replicability of the Policy Codesign process by conducting a walkthrough of the process, providing the intermediary representatives with written, video, and physical artifacts. We will initially send the leaders a project package illustrating the Policy Codesign process, including an overview document outlining the phases and activities and specific examples from each county in story-telling form. This will be supplemented with extracted video recordings of specific activities, pictures of design “artifacts,” and extracts of participant feedback from completed focus groups in written and video form.

We then will hold a focus group organized to elicit feedback on each phase of Policy Codesign. The guide will be informed by the EPIS framework regarding the perceived usability and impact of Policy Codesign as an intermediary intervention that bridges between policy, service sectors, and community (outer setting and inner setting factors) [35].

### Data analysis

#### Aim 1

As the primary aim of the study, analyses are powered with a sample sizeable enough to detect whether the obtained metrics of acceptability and feasibility in this study meet or exceed reasonable standards in the field using noninferiority tests of single proportions. Using sample sizes produced from simulation studies and published by Güllü and Tekindal [53], *n* = 294 is needed to detect an odds ratio difference of at least 0.75. With this sample size, this inferential test can be used to assess perceived policy feasibility and reach (multisector engagement) among the codesign, advisory, and broader community teams (*n* = 400). Measures of acceptability (attendance, satisfaction) among the codesign and advisory teams will be examined as descriptive statistics.

Qualitative (qual) data will be coded using a deductive approach (concurrent, QUANT-qual) and use the six-step triangulation method [54, 55] outlined by Farmer et al. [56] to (a) sort data, (b) code convergence, (c) assess convergence, (d) compare completeness, (e) use researcher comparison, and (f) obtain feedback from the project team and community sites. The primary hypothesis for Aim 1 is that Policy Codesign is an acceptable method that produces feasible adolescent SUD policies, and our analyses aim to identify whether the approach has conceptual or implementation problems that would make replication and testing infeasible.

#### Aim 2

Data will be downloaded from Qualtrics at baseline (T1) 6 months (T2) and 12 months (T3) and analyzed using R package “igraph” (version 1.2.6). The network structure from the initial survey (T1) will provide a baseline for analyzing change at the subsequent timepoint. We expect to see steady increases in network size, connectivity (bridging/brokering activities), and cohesion over the 12 months following the start of the Policy Codesign intervention in both counties. Non-independent inferential analyses and paired *t*-tests will be used to test for changes in these descriptive statistics from baseline to 6 months, and 6 months to 12 months.

We also will use qualitative data to assess stakeholder perceptions of resources necessary for the policy initiative and explore processes of knowledge exchange and integration over time. We will use ATLAS.ti, a computer-assisted qualitative data analysis software, to label participant responses and facilitate the identification of themes. A team of two coders will review textual data and ATLAS.ti labels to distill the labels into overarching themes. This process will be repeated at T1, T2, and T3. We will then engage a side-by-side comparison of themes from T1, T2, and T3 in order to identify points of convergence and divergence over time [57].

#### Integration of findings

Farmer et al.’s [56] triangulation protocol was developed to assess contextual factors affecting health promotion implementation in regional governments with multisector informants and fits well with our aim to capture multiple views about the acceptability and perceived short-term impact of policy codesign. All interviews will be digitally recorded, transcribed, and checked for accuracy by interviewers [58–60]. All interview transcripts and interviewer/moderator notes summarizing interview experience will be entered into ATLAS.ti. A data accounting and backup system will be instituted to keep track of, and facilitate access to, all electronic and hard-copy data. To ensure scientific rigor and credibility of findings, all interviews will be reviewed by two or more members of the research team. When possible, study results will be presented to study participants, enabling them to comment on results and suggest modifications or additional avenues of investigation. Disagreements in the assignment or description of codes will be resolved through discussion among investigators and enhanced definition of codes.

#### Aim 3

We will assess Policy Codesign usability by capturing leaders’ views on its *relative advantage* to other multisector collaboration methods, *ease of use, training or support* needed prior to implementing components, and *motivation* to use it as a strategy. We will record and transcribe focus group(s) and import into ATLAS.ti for analysis. We will use content coding [61] within a deductive framework to identify divergent and convergent themes relative to leaders’ perceptions of usability and impact. This will be done by coding responses first for positive or negative perceptions within usability and impact constructs. We then will use open coding to identify themes in the explanations provided for differing views. Two investigators will develop a codebook after separately coding and then comparing responses for 1/5th of the transcripts. They then will train project research staff to code the interviews until coding reaches 100% agreement after which research staff will code independently. The lead investigators then will review the final, coded transcript to confirm coding accuracy.

## Discussion

This study will address a number of knowledge gaps in the field of policy-focused implementation science, and broader literatures on policy codesign and policymaking. First, the approach draws from theories of public policy and design thinking to anticipate downstream policy implementation problems in the policy formation stage. Existing research on methods to increase the uptake of SUD research evidence in policy focuses heavily on communication strategies (e.g., policy briefs, brokered conversations, forums, and training) [13, 62, 63] and resulting use of research evidence in policy formation. However, the field currently lacks information about methods of policy formation that also advance successful policy implementation.

This study also will be important in that it examines an approach of considerable interest to real-world policy and system professionals, and to behavioral health policy and health design intermediary organizations in and outside of Washington State. The interest is due, in part, to the potential of Policy Codesign to reconcile the demands on the government to model democratic and participatory governance processes while also stewarding the responsible use of public funds. The rapid proliferation of codesign strategies in nonprofit and government sectors is evidence of the need to systematically study efforts to codesign public policies in behavioral health [31]. This study will engage in a systematic study of some of the claims made by codesign practitioners, including ourselves, to inform the wider field about participatory approaches to policymaking in behavioral health [31, 42].

Finally, this study examines the use of creative approaches to translate the research evidence base as a strategy for making this information source more responsive to complex policy contexts. The study will add to the implementation science literature by building on prior work applying the EPIS framework to policy and identifying processes, determinants, and mechanisms likely to impact policy implementation [38]. Rapid evidence synthesis is gaining traction in the scholarly literature as a method to more quickly inform policymakers about research trends and scientific consensus, and is built into the Policy Codesign process [64]. This study adds to this literature by testing design-informed methods of rapid evidence communication [65]. The study will also provide preliminary evidence of acceptability and the perceived feasibility of adopting these translational strategies among well-established behavioral health policy and health design organizations. Overall, results have the potential to impact multiple fields including policy and implementation science, adolescent behavioral health and SUD treatment and prevention, and the treatment/prevention of many other public health problems affecting persons of all ages in the USA.

## Data Availability

Not applicable.

## Abbreviations

SUD: Substance use disorder
EPIS: Exploration, Preparation, Implementation and Sustainment Framework
SNA: Social network analysis
